# Sequential manifestation of Kaposi’s sarcoma and diffuse large B-cell lymphoma in the context of HIV infection: Case report of a rare presentation

**DOI:** 10.1097/MD.0000000000048461

**Published:** 2026-04-24

**Authors:** Yipaer Maimaiti, Silvere D. Zaongo, Gang Bao, Ainiwaer Wulamu, Yaokai Chen

**Affiliations:** a Department of Infectious Diseases, The Sixth People’s Hospital of Xinjiang Uygur Autonomous Region, Urumqi, China; b Department of Infectious Diseases, Chongqing Public Health Medical Center, Chongqing, China.

**Keywords:** AIDS-defining malignancies, diffuse large B-cell lymphoma, HIV, immune dysregulation, Kaposi’s sarcoma

## Abstract

**Rationale:**

Kaposi sarcoma (KS) and diffuse large B-cell lymphoma (DLBCL) are AIDS-defining malignancies frequently linked to HIV-related immune dysregulation. Their sequential occurrence in a single patient is an uncommon and clinically significant phenomenon.

**Patient concerns:**

Herein, we report a case of sequential occurrence of KS and DLBCL that underscores the complex interplay between HIV infection, immune suppression, and oncogenesis.

**Diagnoses:**

At the time of HIV diagnosis (which prompted the immediate initiation of antiretroviral therapy), the patient, a 46-year-old man, exhibited symptoms (generalized rash, bowel movements) raising clinical suspicion for KS. This was confirmed through clinical examinations, including skin biopsy and colonoscopy.

**Interventions:**

The patient received treatment with paclitaxel and doxorubicin for KS. One year later, he developed DLBCL, presenting with back and flank pain, and was subsequently treated with the R-CHOP regimen.

**Outcomes:**

The patient demonstrated a favorable therapeutic response to both treatment regimens.

**Lessons:**

This case underscores the clinical challenges and importance of vigilance in managing sequential AIDS-defining malignancies. It also highlights the need for systematic guidelines for screening oncogenic viruses in HIV-infected individuals, particularly those with prior KS.

## 1. Introduction

Kaposi’s sarcoma (KS) and diffuse large B-cell lymphoma (DLBCL) are distinct and specific malignancies that are strongly associated with human immunodeficiency virus (HIV) infection.^[1]^ Both these conditions are categorized as AIDS (acquired immunodeficiency syndrome)-defining illnesses, and are known to be associated with underlying immune dysfunction and chronic viral co-infections, particularly human herpes virus 8 (HHV-8)^[2]^ and Epstein–Barr virus (EBV),^[3]^ respectively. Despite being relatively common among people living with HIV (PLWH),^[4]^ the simultaneous or sequential presentation of KS and DLBCL in a particular patient is extremely unusual, posing unique challenges to clinical diagnosis, management, and treatment optimization.

Recent publications have reported that KS and DLBCL may co-occur in immunocompromised HIV-negative individuals, most often in older adults.^[5-7]^ This case report highlights a rare occurrence of sequential KS and DLBCL in a relatively young patient (<60 years old) living with HIV. This case is noteworthy in that the patient first developed KS which was successfully treated, and subsequently presented with DLBCL. The striking feature of this report is the exceptional rarity of 2 distinct tumors arising sequentially in the same (relatively younger) individual living with HIV, particularly in the modern era of widely available and highly effective antiretroviral therapy (ART). More importantly, this case shows that lymphoma can develop in individuals living with HIV, even when immune reconstitution has been well achieved. Through detailed clinical evaluation and pathological findings, we explore the interplay between the 2 malignancies, the potential immunological mechanisms at play, and the implications for patient management. This report underscores the importance of vigilance and comprehensive care in HIV-associated malignancies, particularly in cases with atypical presentation, to improve patient outcomes and enhance our understanding of oncogenesis in the context of HIV. Notably, this case emphasizes the clinical need for systematic oncogenic virus screening in patients with HIV who already have KS, a recommendation not explicitly addressed in the prior reports.

## 2. Case presentation

In October 2022, a 46-year-old man of Uyghur ethnicity was admitted to The Sixth People’s Hospital of Xinjiang Uygur Autonomous Region after presenting with a generalized rash. The rash appeared initially on the right lower leg and progressively spread to the feet, face, and postauricular regions. The lesions emerged as pale red spots, gradually enlarging and evolving into purplish-red, spindle-shaped nodules which were elevated above the skin surface. The nodules ranged in size from approximately 2 cm × 1.5 cm to 0.8 cm × 0.5 cm. Laboratory tests confirmed that the patient was positive for HIV, with a severely depleted CD4+ T-cell count of 48 cells/μL. He was promptly initiated on an ART regimen comprising lamivudine (0.3 g orally, once daily), tenofovir disoproxil fumarate (TDF, 0.3 g orally, once daily), and efavirenz (0.6 g orally, once nocte). During a follow-up visit in late December 2022, a skin biopsy of the lesions was performed. Histopathological examination confirmed a diagnosis of Kaposi’s sarcoma, which was previously suspected.

**Figure 1.**
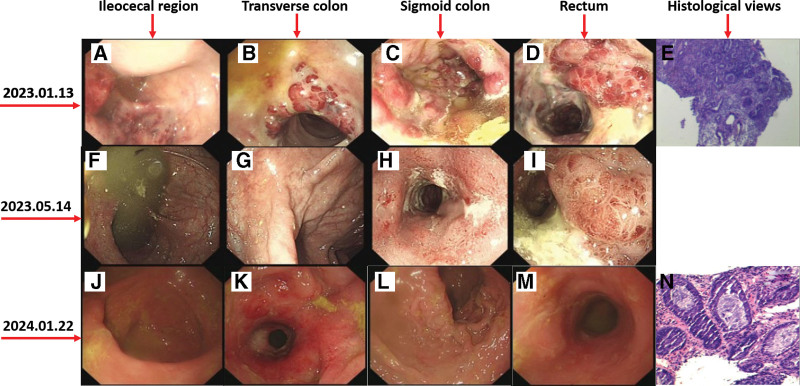
Results from colonoscopy examination on January 13, 2023 (A–E), May 14, 2023 (F–I), and January 22, 2024 (J–O). (A, F, J) Ileocecal region; (B, G, K) transverse colon; (C, H, L) sigmoid colon; (D, I, M) rectum; (E, O) histological views.

In January 2023, follow-up testing revealed a CD4+ T-cell count of 440 cells/μL, indicating a favorable response to antiretroviral therapy (ART). To address potential drug–drug interactions associated with efavirenz and to simplify ART administration, the ART regimen was switched to a once-daily single-tablet regimen of Biktarvy (bictegravir/emtricitabine/tenofovir alafenamide). The patient experienced frequent bowel movements, averaging 10 times/d, along with an unspecified degree of weight loss prior to the ART regimen switch and the symptoms persisted after the switch. A colonoscopy (Fig. 1A–D) and subsequent pathological examinations (Fig. 1E) confirmed a diagnosis of Kaposi’s sarcoma. From January 17 to February 23, 2023, the patient underwent 3 cycles of chemotherapy with paclitaxel injections (150 mg daily). Supportive care was administered concurrently to manage chemotherapy-induced myelosuppression, including interventions to enhance white blood cell and platelet counts. Prior to further intervention, a follow-up evaluation in early March 2023 revealed a remarkable recovery of the CD4+ T-cell count, which reached 742 cells/μL. Notably, at this stage our patient displayed an undetectable HIV viral load (VL < 40 copies/μL). Thus, between March 5 and April 14, 2023, the chemotherapy regimen was transitioned to liposomal doxorubicin hydrochloride injections (32 mg daily via micro-pump) for 3 cycles. This treatment effectively resolved the systemic rash. Follow-up colonoscopies were conducted on May 8, 2023 (Fig. 1F–I), and January 22, 2024 (Fig. 1J–O), providing further evaluation of the patient’s condition. The overall observations indicated a progressive and total remission of KS. Following the remission, we also observed (in January 2024) that the patient’s VL remained undetectable and his CD4+ T-cell count was measured at 665 cells/μL, reflecting substantial and sustained immune system recovery.

**Figure 2.**
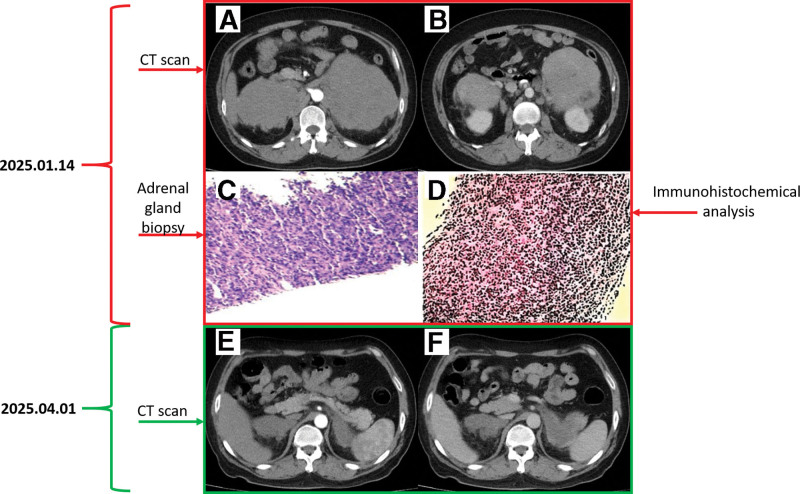
DLBCL diagnosis (A–D) and treatment progression (E, F) as observed on January 14, 2025 and April 1, 2025, respectively. (A, B) Figures show upper abdominal CT scan with contrast before treatment. (C) Figure represents the histological view of adrenal gland biopsy. (D) Figure shows the immunohistochemical findings: CD20 positive (+), CD3 negative (−), Ki-67 positive (60%), and AE1/AE3 negative (−), which are consistent with a diagnosis of B-cell lymphoma. However, due to limited biopsy tissue, precise subtyping is challenging. (E, F) Figures show upper abdominal CT scan with contrast after treatment. CD = cluster differentiation, CT = computed tomography, DLBCL = diffuse large B-cell lymphoma.

**Figure 3.**
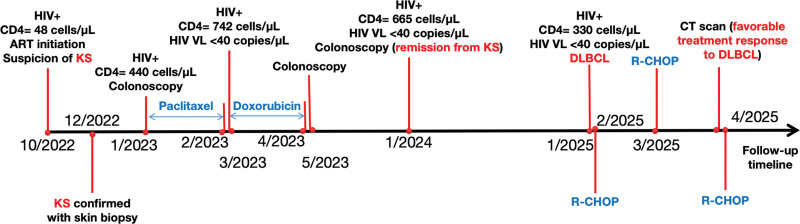
Timeline of the case management. Red lines represent key timepoints, the text in blue refers to chemotherapeutic interventions, and the text in red refers to malignancies observed in the patient. CD = cluster differentiation, CT = computed tomography, DLBCL = diffuse large B-cell lymphoma, KS = Kaposi’s sarcoma.

On January 7, 2025, the patient presented to our hospital again, with complaints of back and waist pain. A contrast-enhanced abdominal computed tomography (CT) scan (Fig. 2A, B) and an adrenal gland biopsy (Fig. 2C, D) indicated and then confirmed a diagnosis of diffuse large B-cell lymphoma (DLBCL). At the time of presentation, the patients had a CD4+ T-cell count of 330 cells/μL and an undetectable HIV viral load; however, EBV was not tested for at this stage. Chemotherapy commenced on February 24, followed by additional cycles on March 14 and April 3, 2025, utilizing the R-CHOP regimen, which included Rituximab (day 0), Cyclophosphamide (day 1), Doxorubicin (day 1), Vincristine (day 1), and oral prednisone (100 mg daily, days 1–5). Supportive care was provided throughout treatment and included liver protection, antiemetics, antiallergy medications, and urine alkalization. These measures contributed to a significant alleviation of the patient’s back and waist pain. A follow-up contrast-enhanced CT scan of the upper abdomen conducted on April 1, 2025 (Fig. 2E, F) demonstrated favorable treatment response, and provided further guidance for ongoing management. A summary of this case and its overall management is presented in Fig. 3.

## 3. Discussion

Here, we present a rare and noteworthy case of an HIV-infected patient who sequentially developed KS and DLBCL in the present era of modern ART. The underlying mechanisms driving this unusual progression remain challenging to explain; however, this uncommon presentation is likely to stem from the complex and dynamic interactions among HIV, immune suppression, and the presence of preexisting latent oncogenic viruses in this patient.

KS is a well-recognized AIDS-defining malignancy associated with HHV8.^[8]^ At initial presentation, our patient exhibited significantly low CD4+ T-cell counts, favoring a permissive environment for the development of KS. While the direct presence of HHV8 was not confirmed in this case, the diagnosis of KS was established based on findings from a skin biopsy and colonoscopy. Encouragingly, the initiation of ART and appropriate chemotherapy for KS led to substantial clinical improvement, enabling the patient to recover from KS and achieve robust immune restoration, with CD4+ T-cell counts improving from 48 cells/μL in October 2022 to 665 cells/μL in January 2024. Unexpectedly, the subsequent emergence of DLBCL coincided with a significant decline in CD4+ T-cell counts, dropping from 665 cells/μL in January 2024 to 330 cells/μL in January 2025. This perplexing sequence raises critical questions regarding the fundamental mechanisms driving this clinical trajectory.

A review of the literature reveals that patients with KS, particularly those coinfected with HIV, face an elevated risk of developing non-AIDS-defining malignancies.^[9,10]^ For example, Shiels et al^[10]^ have shown that individuals with HIV who develop KS have a 4.6-fold higher risk of experiencing additional malignancies. However, the simultaneous or sequential occurrence of KS and DLBCL is uncommon and rare. This phenomenon may be attributable to the chronic inflammation and shared immune suppression mediated by HIV and oncogenic viruses such as HHV8 (associated with KS) and Epstein–Barr virus (EBV, linked to DLBCL). In this case, the presence of EBV was not investigated and the patient was negative for HBV and HCV. However, it is well-established that, besides EBV, other oncogenic viruses such as HIV, HHV8, hepatitis B and C viruses, and human T-cell lymphotropic virus-1 (HTLV-1) may be associated with the development of DLBCL.^[11-14]^ In this particular case, it is plausible either that another virus may have contributed to the onset of the observed condition, or that HIV alone could have been a factor adequate enough to instigate the emergence of DLBCL. A more comprehensive profiling of potentially oncogenic viruses at the time of DLBCL diagnosis in this patient would have been crucial in elucidating the true etiology of the disease. That said, the overwhelming immunosuppressive effects of HIV-associated immunopathology, particularly its role in the promotion of chronic inflammation and immune suppression, are well documented.^[15]^ In this case, however, the undetectable HIV VLs observed in our patient since March 2023 suggests that HIV is unlikely to be the primary cause of the progressive immune suppression observed. Interestingly, it is known that HHV8,^[16]^ EBV,^[17]^ and HTLV-1^[18,19]^ may exacerbate immune dysregulation, contributing to persistent immune suppression. In March 2023, during the course of KS treatment, the patient was observed to have a CD4+ T-cell count of 742 cells/μL. By January 2024, following the completion of KS treatment, this count had stabilized at 665 cells/μL. However, the subsequent decline to 330 cells/μL by January 2025, despite continued adherence to an effective modern ART regimen, underscores the potential persistence of underlying mechanisms driving ongoing CD4+ T-cell depletion. This immune suppression may result from the synergistic inhibitory effect of HIV and oncogenic viruses, in spite of the presence of effective ART. While ART initially restored CD4+ T-cell counts during the early period of the patient’s illness (from 2023 to 2024), functional immune deficits (such as Th1/Th2 imbalances), reduced γ-interferon production, and impaired antiviral responses may have rendered the patient vulnerable to latent oncogenic viruses that the patient had previously been exposed to. These viruses, in turn, may have exacerbated immune suppression, as reflected by the precipitous CD4+ T-cell decline observed in 2025. This complex immunopathological interplay warrants further investigation in order to elucidate the fundamental underlying mechanisms that drive the clinical processes and outcomes observed in our patient. Understanding these dynamics will be crucial for optimization of therapeutic strategies for patients having HIV infection in conjunction with concurrent latent or overt oncogenic virus infections.

Given the heightened risk of individuals with HIV and comorbid KS developing subsequent malignancies such as DLBCL, continuous and vigilant monitoring is imperative to facilitate early detection and improve treatment outcomes. In our case, the diagnosis of DLBCL occurred 1 year following the treatment of KS, with the patient fortunately achieving a favorable response to DLBCL treatment after total remission of KS. However, this timeline underscores the potential for rapid clinical deterioration within a relatively short period. To enhance the likelihood of early detection and timely intervention, we advocate for more frequent follow-up visits for patients with a history of KS. Annual clinical follow-up assessments may be insufficient given the potential progressive nature of these conditions. Shorter intervals between medical evaluations may prove critical in ensuring more robust prognoses and in mitigating the risks associated with delayed diagnoses. More importantly, a systematic evaluation of known oncogenic viruses should be undertaken in patients who have already developed KS in order to enhance the monitoring and long-term management of these patients.

## Author contributions

**Conceptualization:** Yipaer Maimaiti, Silvere D. Zaongo.

**Supervision:** Yaokai Chen.

**Writing – original draft:** Yipaer Maimaiti, Silvere D. Zaongo.

**Writing – review & editing:** Yipaer Maimaiti, Silvere D. Zaongo, Gang Bao, Ainiwaer Wulamu, Yaokai Chen.
